# Misinformation About Medical Cannabis in YouTube Videos: Systematic Review

**DOI:** 10.2196/76723

**Published:** 2025-10-06

**Authors:** Shivank Khare, Simon Erridge, Swathikan Chidambaram, Mikael Hans Sodergren

**Affiliations:** 1 Department of Surgery and Cancer, Academic Surgical Unit, Imperial College London, 10th Floor QEQM, St Mary’s Hospital, South Wharf Road, London, W2 1NY, United Kingdom, 44 203 312 6666

**Keywords:** cannabis, consumer health information, health literacy, health education, health promotion

## Abstract

**Background:**

YouTube has become a major source of health information, with 2.5 billion monthly users. Despite efforts taken to promote reliable sources, misinformation remains prevalent, particularly regarding medical cannabis.

**Objective:**

This study aims to evaluate the quality and reliability of medical cannabis information on YouTube and to examine the relationship between video popularity and content quality.

**Methods:**

A systematic review of YouTube videos on medical cannabis was conducted. Search terms were selected based on Google Trends, and 800 videos were retrieved on July 8, 2024. After applying exclusion criteria, 516 videos were analyzed. Videos were categorized by content creators: (1) nonmedical educational channels, (2) medical education channels, and (3) independent users. Two independent reviewers (SK and SE) assessed content quality using the DISCERN grade and the Health on the Net (HON) code. Statistical analysis included one-way ANOVA and Pearson correlation coefficient.

**Results:**

Of the 516 videos analyzed, 48.5% (n=251) were from the United States, and 17.2% (n=89) from the United Kingdom. Only 12.2% (n=63) were produced by medical education channels, while 84.3% (n=435) were by independent users. The total views reached 119 million, with nonmedical educational channels having the highest median views with 274,957 (IQR 2161-546,887) and medical education channels having the lowest median views at 5721 (IQR 2263‐20,792.50). The mean DISCERN and HON code scores for all videos were 34.63 (SD 9.49) and 3.93 (SD 1.20), respectively. Nonmedical educational creators had the highest DISCERN score (mean 47.78, SD 10.40) and independent users had the lowest score (mean 33.5, SD 8.50; *P*<.001). Similarly, nonmedical educational creators had the highest HON code score (mean 5.33, SD 1.22), while independent users had the lowest (mean 3.78, SD 1.10; *P*=.007). Weak positive correlations were found between video views and DISCERN scores (*r*=0.34, *P*<.001) and likes and DISCERN scores (*r*=0.30, *P*<.001).

**Conclusions:**

YouTube is a key source of information on medical cannabis, but the credibility of videos varies widely. Independent users attract the highest viewers but have reduced reliability according to the DISCERN and HON scores. Educational channels, despite increased reliability received the least engagement. The weak correlation between views and content quality emphasizes the need for content moderation to ensure that the most reliable and accurate information on health issues is widely disseminated. Future research should identify strategies to promote verified sources of information and limit misinformation.

## Introduction

The therapeutic use of cannabis has undergone a dramatic transformation in public perception and legal status over the past decade. As such, cannabis is now one of the most consumed drugs. According to the 2014 National Survey on Drug Use and Health, cannabis was the most used illicit substance in the United States, with 13.2% of individuals aged ≥12 years reporting cannabis use in the past year and 8.4% in the past month [1]. Similar trends are also seen in Europe, Asia, and Australasia [2-4]. This shift reflects both evolving legislative frameworks and growing public acceptance of medical cannabis [5]. However, the rapid normalization of medical cannabis has outpaced the generation of high-quality evidence on its efficacy and safety. Current randomized controlled trials are highly heterogenous, limited by small sample sizes and use short follow-up assessments [6]. Consequently, there is a paucity of clinical guidance and education available to inform clinicians and their patients about medical cannabis. Within this context, YouTube—as the world’s second-most popular social media network [7]—has become a key platform for distributing health-related information and shaping patient understanding of this class of medications.

The democratization of health information through social media platforms presents unique challenges for medical professionals and public health authorities. Unlike peer-reviewed literature or guidance from clinical associations, YouTube’s algorithms prioritize engagement metrics over accuracy, potentially amplifying sensationalist or commercially motivated content. A study of COVID-19 misinformation on YouTube highlighted that misleading information was least prevalent in professional or government videos; however, these had lower views and engagement [8]. In response to misinformation propagated during the COVID-19 pandemic, YouTube has implemented community guidelines to help mitigate medical misinformation. These guidelines classify misinformation into three categories: disease prevention, medical treatments, and denial of specific health conditions or their consequences in accordance with the World Health Organization (WHO) [9]. Despite these regulatory efforts, misinformation remains prevalent on YouTube, indicating an ongoing challenge in producing accurate and reliable medical information.

Medical cannabis has emerged as a topic of considerable interest on YouTube, fueled by growing research into its potential therapeutic applications for various conditions, including pain, anxiety, and other chronic illnesses [10]. Misinformation about medical cannabis can be detrimental to public perception. Studies indicate that news articles and blogs often exhibit a strong positive bias toward the efficacy of cannabinoids in pain management [11]. This may foster unrealistic treatment expectations based on online information. In addition, analysis from the International Cannabis Policy Study highlights a reduced perception of some potential risks associated with cannabis [11]. This raises concerns about potential harm to individuals who may struggle to distinguish between evidence-based data and unverified claims, widening the gap between perceptions and the ground truth.

A prior analysis of YouTube videos highlighted that, out of 66 videos on cannabis for chronic pain, there was a wide range of video quality and reliability. The videos of the highest quality were most likely to be produced by physicians. However, due to the limited sample size and focus on chronic pain, this analysis limits insights into engagement with misleading or harmful videos. Consequently, there is an ongoing paucity of information on the quality and reliability of information on cannabis and health on YouTube and other social media platforms [12]. This review therefore provides a timely assessment of the quality of medical cannabis information available on YouTube. The primary aim is to assess the objective quality of the videos with the highest reach on the platform. In addition, the study aims to investigate the relationship between video popularity and the accuracy and quality of the information presented.

## Methods

A systematic review was performed of the content published on YouTube (Alphabet Inc) on medical cannabis. The conduct and reporting of this study were performed in line with Preferred Reporting Items for Systematic reviews and Meta-Analyses extension for Scoping Reviews (PRISMA-ScR) [13].

### Ethical Considerations

This study involved a retrospective review of publicly accessible YouTube videos and did not involve human participants or identifiable private information. As such, formal ethical approval was not required. This was confirmed by the Imperial College London ethics panel. All data used in the study were publicly available and did not contain any personally identifiable information. Videos were analyzed anonymously, and no attempts were made to contact content creators or link content to specific individuals.

### Search Strategy and Data Extraction

To ensure relevancy of the search strategy for medical cannabis, key terms were derived from an assessment of worldwide Google Trends (Google LLC) data from the 12 months preceding July 8, 2024, to ensure the highest coverage of cannabis-based medicinal products. These included ‘medical cannabis,’ ‘medical marijuana,’ ‘medical weed’ and ‘prescription cannabis.’ These search terms were then entered into the United Kingdom version of YouTube (Alphabet Inc) on July 8, 2024, using incognito mode to prevent third-party cookies and browsing history from affecting the results.

The top 200 videos of each search term were noted using Microsoft Excel spreadsheet with the key attributes: title, number of views, country of origin, duration, age, channel name, number of likes, and number of comments. Exclusion criteria included videos shorter than one minute, older than five years (based on upload date)**,** duplicate videos, not in English, or unrelated to cannabis-based medicinal products, such as those not addressing their biochemistry, effects or social topics.

Data extraction was performed by two independent reviewers (SK and SE), with disagreements planned to be resolved by a third author (SC). Data extraction included video views, likes, channel, and country of origin. The videos were subsequently categorized into the following three categories based on the channel types and purpose: educational channels organized by nonmedical professionals (eg, TED or TEDx Talks); education channels organized by medical professionals (eg, Mayo Clinic or Curaleaf Clinic); and independent nonmedical users and groups (eg, independent content creators).

### Outcome Measures

The primary outcome was the quality and reliability of the video content. This was determined using the DISCERN Grade and Health on the Net (HON) code. These tools have been used previously to assess health care information based on social media influence [14]. The DISCERN Grade consists of 16 questions, with each question scored on a scale of 1 to 5 points, therefore making a total of 80 points. It is divided into three sections, with the first section evaluating the reliability of the paper, the second assessing the quality of information on treatment choices and the final section examining the overall quality of the publication [15]. All sections of the DISCERN grade are included in the sum score. The HON code is used to ensure health and medical websites maintain honesty and serve as reliable sources of information backed up by evidence. There are eight key principles, and each video is scored on a scale from zero to eight, where zero indicates noncompliance, and eight represents full compliance [16]. The videos were assessed independently by SK and SE. Any discrepancies were planned to be resolved through discussion with a senior author (SC). Secondary outcomes included assessment of video reach and engagement through video views and likes.

### Statistical Analysis

The YouTube engagement metrics were summarized using median (IQR), while DISCERN grade scores, and HON code scores were summarized using mean (SD). Statistical analysis was performed using PRISM (version 10; GraphPad software). One-way ANOVA was conducted to compare DISCERN and HON scores between different content creators assuming unequal variances.

Pairwise comparisons were conducted using Tukey test to identify specific differences between groups. The Tukey method was applied for multiple comparisons to control for Type I error and was conducted when a statistically significant finding was present on ANOVA. Pearson correlation coefficient was used to determine the association between the DISCERN Grade and the number of views. Statistical significance was defined as *P* value <.05.

## Results

**Figure 1.**
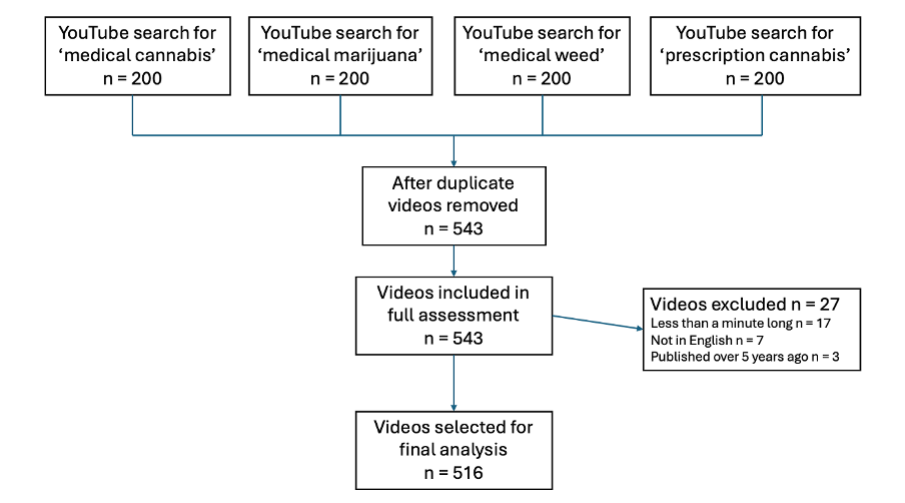
Flow chart showing the video selection process for information pertaining to medical cannabis including the exclusion criteria and video duplicates.

The YouTube platform was searched on July 8, 2024, with the four search phrases yielding 800 videos in total. Two hundred and fifty-seven videos were duplicates and were subsequently removed. A further 27 videos did not meet the inclusion criteria. This led to 516 videos being included in the final analysis (Multimedia Appendix 1). The video search and data extraction can be depicted in the illustration in Figure 1.

Most videos originated from the United States of America (n=251, 48.65%), followed by Europe (n=94, 18.22%). Seventeen (3.29%) videos had more than a million views. Only 12.2% (n=63) of videos were produced by medical professionals, with most videos produced by independent organizations (n=435; 84.3%). The important baseline characteristics are mentioned below in Table 1, including the country of origin, channel type, and engagement metrics.

**Table 1.** Baseline characteristics of YouTube videos (N=516).

Characteristic	YouTube videos (N=516)
Country of Origin, n (%)	
Australia	33 (6.4)
Canada	16 (3.1)
Denmark	2 (0.4)
Germany	3 (0.6)
India	1 (0.2)
Israel	1 (0.2)
Korea	1 (0.2)
Malaysia	1 (0.2)
New Zealand	9 (1.7)
Norway	1 (0.2)
Philippines	4 (0.8)
Qatar	1 (0.2)
Singapore	1 (0.2)
South Africa	5 (1.0)
Sri Lanka	1 (0.2)
United Kingdom	89 (17.2)
United States of America	251 (48.5)
Unknown	96 (18.6)
Channel types, n (%)	
Educational (Nonmedical)	18 (3.5)
Educational (Medical)	63 (12.2)
Independent users	435 (84.3)
Engagement metrics, median (IQR)	
Views	9,605 (4,347-39,029)
Views per day	12 (4-45)
Likes	169 (67-590)

Table 2 presents the engagement metrics and quality assessment indicators for YouTube videos addressing medical cannabis misinformation, with a combined total of 119,155,949 views since upload. Among the three video categories, independent users contributed most of the total views (91,660,510), followed by educational (nonmedical) creators with 23,026,930 views, and educational (medical) sources with 4,468,509 views.

**Table 2.** Engagement metrics, adherence to the HON code of conduct, and DISCERN scores for YouTube videos on medical cannabis misinformation..

Parameter	Educational (Nonmedical) (n=18)	Educational (Medical) (n=63)	Independent users (n=435)	*P* value^a^
Engagement metrics				
Total views, n	23,026,930	4,468,509	91,650,510	—^b^
Views per video, median (IQR)	274,957 (2,161-546,887)	5,721 (2,263–20,793)	10,447 (4,625-38,557)	.001
Likes, median (IQR)	6,063 (28–9,060)	133.5 (49-338)	174 (69-594)	<.001
Quality assessment indicators, mean (SD)				
HON^c^ adherence total score	5.22 (1.22)	4.59 (1.43)	3.78 (1.10)	.007
DISCERN total score	47.78 (10.40)	38.59 (12.73)	33.50 (8.50)	<.001

a *P* values are calculated using one-way ANOVA.

b Not applicable.

c HON: Health on the Net foundation.

When examining views per video, educational (nonmedical) creators had the highest median view number with 274,957 (IQR 2161-546,887) views, substantially outperforming both independent users with 10,447 (IQR 4625-38,557) views and educational (medical) creators with 5721 views (IQR 2263-20,793) views. Post hoc pairwise comparisons revealed that the differences between independent and nonmedical sources (*P*<.001), as well as between medical and nonmedical sources (*P*<.001), were statistically significant. There was no statistically significant difference between independent and medical sources (*P*=.67).

A similar trend was observed for likes, where educational (nonmedical) creators garnered the most engagement with 6063 (IQR 28–9060) likes, followed by independent users with 174 (IQR 69-594) likes, and lastly, educational (medical) creators with 134 (49-338) likes. Tukey multiple comparisons test confirmed that independent sources significantly differed from nonmedical sources (*P*<.001), and medical sources also significantly differed from nonmedical sources (*P*<.001). However, there was no difference between independent and medical creators (*P*=.83).

In terms of quality assessment, educational (nonmedical) videos demonstrated the highest standards, with HON adherence scores averaging 5.22 (SD 1.22) and DISCERN scores at 47.78 (SD 10.40). In comparison, independent users produced content with the lowest quality scores (HON: mean 3.78, SD 1.10; DISCERN: mean 33.50, SD 8.50), while educational (medical) sources occupied a middle ground (HON: mean 4.59, SD 1.43; DISCERN: mean 38.59, SD 12.73). Post hoc testing showed significant differences in DISCERN scores across all groups, including independent versus medical (*P*<.001), independent versus nonmedical (*P*<.001), and medical versus nonmedical sources (*P*<.001). Regarding HON adherence, educational (nonmedical) sources outperformed both independent users (*P*<.001) and medical creators (*P*<.001), though the difference between medical and independent sources was not statistically significant (*P*=.07).

**Figure 2.**
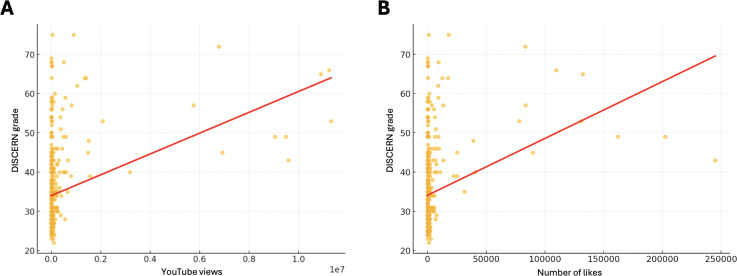
The relationship between engagement metrics and DISCERN grade score. (A) A scatter plot with a positive trend line indicating a weak correlation (0.34) between an increased number of views and DISCERN grade scores. (B) Scatter plot with a positive trend line with a weak correlation (0.30) between increased likes and DISCERN grade scores. Correlations were assessed using Pearson correlation coefficient.

The mean DISCERN and HON code scores across all videos were 34.63, SD 9.49 and 3.93, SD 1.20, respectively. Pearson correlation coefficient analysis revealed a low positive correlation (*r*=0.34; *P*<.001) between YouTube video views and DISCERN scores and a low positive correlation of (*r*=0.30; *P*<.001) between likes and DISCERN scores (Figure 2).

## Discussion

### Principal Findings

This study aimed to evaluate the quality of information presented in YouTube videos about medical cannabis and assess whether popularity correlates with content reliability. The findings reveal a notable disconnect between viewer engagement and video quality, with independent users gathering the highest views but offering the least reliable content, as indicated by lower DISCERN and HON scores. These results emphasize the persistent challenge of misinformation on digital platforms, even in an era of increasing regulation.

The overall mean DISCERN score across all videos was 34.63, which, on the 80-point scale, suggests generally poor reliability. In comparison, other studies examining health topics such as varicoceles and testicular torsion exhibited similar concerns [17,18]. The analysis by Hong et al [18] expressed frustration as from the nonduplicated videos, an overwhelming majority were expressed to be educational but were rated as poor on the DISCERN score. A comparable pattern was observed in the present analysis with only 12.2% of the videos originating from medical or expert sources, with the overall mean DISCERN score. This suggests that misinformation about medical cannabis is not an isolated issue but part of a broader trend of unreliable health communication online.

A noticeable finding in this study was videos from medical education sources were less popular and had lower quality and reliability scores in comparison to nonmedical educational sources. This is a challenge also seen in other areas of health content on YouTube [19]. One reason could be medical sources often use technical language and longer video formats suited for academic purposes but less engaging to the public. In contrast, nonmedical creators may adopt language and entertaining visuals, which enhances the relatability and shareability for lay audiences. Moreover, while the quality and reliability of medical videos was higher than independent creators, it was lower than that of the nonmedical educational content. Many of the nonmedical sources were from news organizations; therefore, the introduction of clear editorial policies for medical channels may help further enhance their quality. While independent creators received lower DISCERN and HON code scores, they had higher engagement. Evidence demonstrates that subjective language and content that falls under entertainment categories are more likely to receive a higher number of views and likes [20]. In addition, posting outside of business hours is more likely to receive higher engagement. Independent creators are less bound by professional standards of objectivity and are also more likely to focus on changing posting frequency and timings to maximize video engagement. YouTube has introduced initiatives to try and help improve the reach of content produced by accredited health care professionals. It is also important that this content continues to be as engaging and meets viewers at their level for effective dissemination of health information on this particular social media platform [21].

Furthermore, a study on COVID-19 vaccine videos found that approximately 11% of the most-viewed videos contradicted the reference standards from the WHO or the Centers for Disease Control and Prevention. These videos had significantly lower modified DISCERN (mDISCERN) scores compared to factual ones, yet they garnered far more likes and engagement [22]. Similarly, this analysis identified several of the most viewed videos were of cannabis content published by nonexpert independent users with low reliability.

The weak positive correlation between engagement metrics (views and likes) and DISCERN scores (*r*=0.34 and *r*=0.30, respectively) further confirms that popular videos are not necessarily trustworthy. Moreover, visual analysis of correlation plots highlights that the r value is likely impacted by several outlier videos. This is consistent with research by Stimpson and Ortega [23], which found that a large proportion of social media users (82%) perceive false or misleading health information to be prevalent on these platforms, with 67% admitting they are unable to assess the accuracy of the information they encounter [23]. This increases the risk of misinformed health decisions by patients and the general public, but may also increase anxiety among viewers [24]. Videos that over illustrate the therapeutic benefits of cannabis while downplaying risks, such as adverse effects or drug interactions, may lead individuals to experiment with unproven treatments without medical guidance. For example, some cancer patients believe cannabis can treat their cancer directly, despite a lack of scientific evidence, potentially leading to worse outcomes if they prioritize unproven cannabis use over chemotherapy or immunotherapy [25].

YouTube enforces strict policies on cannabis-related content, prohibiting videos that display or promote the consumption of cannabis and typically permitting educational, documentary, or news-focused discussions if they do not encourage use or provide instructions for consumption [26]. While YouTube’s approach to misinformation prohibits health claims that contradict recognized authorities and removes content with serious risk of harm, the platform does not systematically check creators for medical credentials or require source acknowledgment, resulting in only partial alignment with the more stringent transparency and authority requirements of the HON code.

To combat the spread of misinformation, the WHO is working with the Council of Medical Specialty Societies and the National Academy of Medicine on Phase II of a project on identifying credible sources of health information on social media [27]. The project aims to provide social media platforms with standardized criteria to distinguish reliable health information, addressing challenges like inconsistent vetting processes and the proliferation of unverified claims. However, systemic issues like algorithmic bias and regulatory inertia continue to remain unresolved.

Several limitations of this study should be acknowledged. First, the video selection was restricted to English-language content, which limits the generalisability of the findings to non-English speaking populations and other geographic regions. Additionally, while the DISCERN tool provides a structured framework for assessing information quality, it allows for subjective interpretation, particularly when assigning partial scores between 2 and 4. This introduces some variability in how each criterion is evaluated. The study also did not consider channel-level data or account characteristics, which may have implications for both the reach and perceived credibility of the videos hosted on that channel. Lastly, this study focused solely on YouTube and did not examine other social media platforms such as Instagram, TikTok, or Facebook, which are also social media sites where individuals may be exposed to unverified health information.

### Conclusion

In conclusion, the quality of information about medical cannabis among the most viewed videos on YouTube remains poor. This mirrors similar patterns observed during the COVID-19 pandemic and in studies of other health topics. This study contributes to a growing body of evidence suggesting that user engagement does not strongly correlate with informational quality, posing ongoing challenges for public health communication. In the future, it is important YouTube and other social media sites implement policy changes to mitigate the spread of misinformation in all fields, particularly health, and to regulate the content by health care professionals more frequently. Medical professionals should also be encouraged to engage more actively on social platforms and implement strategies to make their content engaging as well as informative to compete with low-quality content.

## Supplementary material

10.2196/76723 Multimedia Appendix 1 516 YouTube videos included in the final analysis.

10.2196/76723 Checklist 1 PRISMA checklist.

## Abbreviations

HON: Health on the Net
PRISMA-ScR: Preferred Reporting Items for Systematic reviews and Meta-Analyses extension for Scoping Reviews
WHO: World Health Organization
